# Optical chemosensors for environmental monitoring of toxic metals related to Alzheimer's disease†

**DOI:** 10.1039/d2ra05384e

**Published:** 2022-11-15

**Authors:** Islam M. El-Sewify, Ahmed Radwan, Nehal H. Elghazawy, Wolfgang Fritzsche, Hassan M. E. Azzazy

**Affiliations:** Department of Chemistry, Faculty of Science, Ain Shams University 11566 Abbassia Cairo Egypt; Department of Chemistry, School of Sciences & Engineering, The American University in Cairo SSE, Rm #1194, P.O. Box 74 New Cairo 11835 Egypt hazzazy@aucegypt.edu; Department of Nanobiophotonics, Leibniz Institute for Photonic Technology Jena 07745 Germany

## Abstract

Alzheimer's disease (AD) is the most common type of dementia and progresses from mild memory loss to severe decline in thinking, behavioral and social skills, which dramatically impairs a person's ability to function independently. Genetics, some health disorders and lifestyle have all been connected to AD. Also, environmental factors are reported as contributors to this illness. The presence of heavy metals in air, water, food, soil and commercial products has increased tremendously. Accumulation of heavy metals in the body leads to serious malfunctioning of bodily organs, specifically the brain. For AD, a wide range of heavy metals have been reported to contribute to its onset and progression and the manifestation of its hallmarks. In this review, we focus on detection of highly toxic heavy metals such as mercury, cadmium, lead and arsenic in water. The presence of heavy metals in water is very troubling and regular monitoring is warranted. Optical chemosensors were designed and fabricated for determination of ultra-trace quantities of heavy metals in water. They have shown advantages when compared to other sensors, such as selectivity, low-detection limit, fast response time, and wide-range determination under optimal sensing conditions. Therefore, implementing optical chemosensors for monitoring levels of toxic metals in water represents an important contribution in fighting AD.

## Abstract 2

This review briefly summarizes evidence that links toxic metals to onset and progression of Alzheimer's disease. It discusses the structure and fabrication of optical chemosensors, and their use for monitoring toxic metals in water.

## Graphical abstract

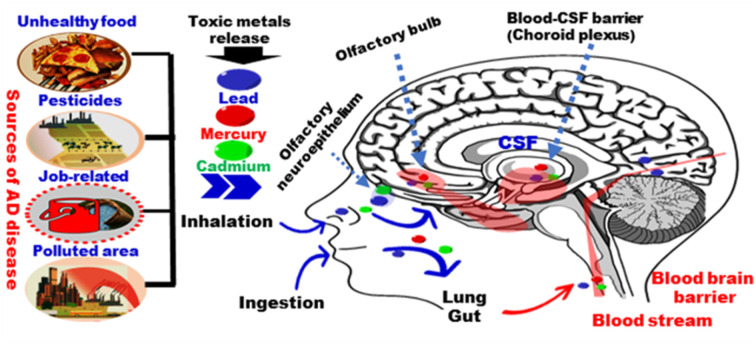

## 1. Introduction

Dementia, a neurodegenerative disorder, is characterized by loss of memory, language and problem-solving along with other thinking abilities, which can interfere with a patient's daily life. Dementia can be manifested in different formats such as Alzheimer's Disease, vascular dementia, and Lewy body dementia.^1^ Alzheimer's disease (AD) is known to be the most common form of dementia where according to the WHO it represents 60–70% of dementia cases worldwide.^2^ Although AD is commonly diagnosed in aged societies that are older than 65 years old, some cases are considered “early-onset” where symptoms are witnessed as early as 30 years old. For diagnosis, AD is known to have very distinct hallmarks represented in the intracellular neurofibrillary tangles (NFTs) containing the protein tau in a hyper-phosphorylated state and the extracellular plaques containing amyloid beta (Aβ).^3–6^ Regardless the distinctive diagnostic markers for AD, unfortunately, the causes behind AD are not quite understood up till now. As such, studying the etiology of AD has been quite a challenging task where it resulted in declaring AD as a multifactorial disorder. Generally, the increase in the incidence of AD along with its progression has been attributed to genetics, environmental factors, and acquired factors such as cerebrovascular diseases, stress, anxiety, and sleep disorders. It can be quite understandable that some risk factors are hardly controllable, still, identifying the controllable ones has become paramount to reduce the AD progression.^7,8^ Latest observations have identified environmental risk factors as key causal players in the progression and onset of AD. Those factors including air pollution, poor diet, contaminated water, and infections can participate in developing AD by initiating oxidative stress and inflammation.^9,10^ Among the well-known environmental risk factors is the prolonged exposure to heavy metals especially those of industrial origin. Such pollutants can be released in the air as fine dust or in water as well as the soil. Those heavy metals have the capacity to initiate the formation of Aβ plagues and the phosphorylation of the tau protein; thereof, neuronal cell death is a definite outcome.^11^ In this review, we give a special attention to those heavy metals and how to detect them in water, in hope that these measures act as a protective shield against AD.

## 2. Heavy metals and Alzheimer's disease

**Fig. 1:**
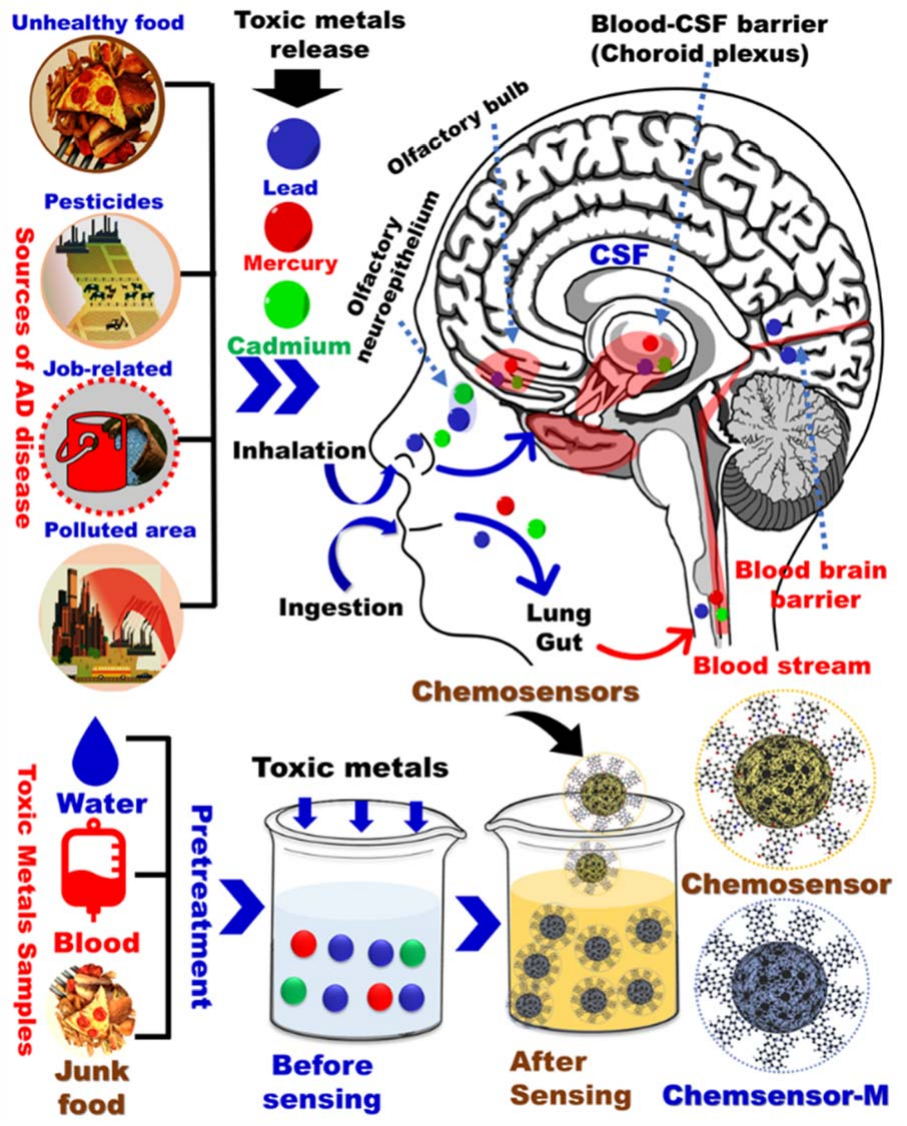
General schematic diagram of common environmental factors associated with Alzheimer's disease.

Heavy metal poisoning is an anticipated consequence for the excessive exposure to heavy metals through many routes such as air, water, improperly coated food containers, and industrial exposure. While all those routes are critical, water contamination can be of the highest incidence as well as the largest impact. Accordingly, understanding the water contamination and the available techniques for its detection is essential. Although many heavy metals such as lead (Pb), mercury (Hg), cadmium (Cd), chromium (Cr), copper (Cu), zinc (Zn), iron (Fe), manganese (Mn), and arsenic (As) have shown some contribution to AD,^12,13^ we herein focus on the highly toxic ones *i.e.* Hg, Cd, Pb, As (Fig. 1). Upon accumulation in the body, these metals contribute to the AD progression as illustrated below. Various agencies and organizations such as the World Health Organization (WHO), European Union (EU), and the US Environmental Protection Agency (US EPA) have established drinking water guidelines for heavy metals in water (Table S1†).

### 2.1. Mercury

Mercury (Hg) can exist in elemental state, ionic state, and organic form (methyl mercury). Mercury is introduced into the environment as a result of volcanic emissions or industrial activities. Since the industrial revolution, the amount of mercury in both air and water has increased by 3–5 folds.^14–16^ Hg is a potent neurotoxin that causes cognitive and motion disorders. It has been shown to affect AD onset and progression *via* multiple routes. Mercury was reported to (a) stimulate the formation of beta-amyloid protein causing senile plaques;^17–19^ (b) increase the phosphorylation of tau protein resulting in NFTs;^20^ (c) inhibit glutamate uptake and stimulate its release causing neurodegeneration in AD;^21^ and (d) interfere with some enzymes related to AD such as beta-amyloid cleaving enzyme BACE-1, monoamine oxidase, and acetylcholine transferase.^22–24^ Surprisingly, Hg was also linked to the genetic component of AD where it causes presenilin1 gene mutation which is linked to 30% of early-onset AD cases.^25^ Also, research on rat model has shown that neuronal beta-tubulin defects may be induced by inorganic mercury proposing that Hg may interfere with the assembly of microtubules from tubulin that eventually leads to plaques.^26,27^ In humans, Hg concentration in the plasma of AD patients was almost twice the values from age matched controls.^28^ Also, workers exposed to Hg vapors for long time, showed signs of cognitive deficits.^29,30^ Finally, studies on brain autopsies of AD patients showed Hg deposition in the hippocampus and amygdala, which are involved in memory and other cognitive functions.^31,32^

### 2.2. Cadmium

Cadmium (Cd) is a toxic water-soluble metal characterized by a long half-life in humans where it accumulates in the body, especially in the kidneys, liver, lung, and brain and can induce several toxic effects, depending on its concentration and exposure time. Cd is a carcinogen with a certain ability to cross the blood–brain barrier and has been linked to neurological diseases such as AD. Cd pollution of water, air, soil and food arises from cigarette smoking and several industries such as nickel–cadmium batteries, pigments, plastic stabilizers, and metal refineries.^33–35^ Several studies reported the interference of Cd with some key factors related to AD. Cd is able to interact with beta amyloid proteins inducing their aggregation which is a distinctive hallmark for the pathogenesis of AD.^33,36,37^ It also downregulates the expression of α-secretase (ADAM10) and neutral endopeptidase which play important roles in reducing Aβ levels in the brain.^38^ Cd may be involved in self-aggregation of tau in the AD brain and selectively blocks M1 receptors that help in the negative regulation of GSK-3β (an enzyme when overexpressed increase both total and phosphorylated tau protein).^39,40^ Additionally, Cd was reported to activate mitogen-activated protein kinase (MAPK) and NF-κB signaling pathways which elevate the levels of IL-6 and IL-8 that are associated with AD pathogenesis.^41,42^ Unfortunately, there has been difficulty in assessing the effects of Cd *in vivo* or in AD patients, since metal binding proteins, stress proteins and antioxidants may modulate the toxicity of the element. Still, in an *in vivo* study, APP/PS1 transgenic mice which consumed to drinking water contaminated with Cd exhibited an increase in the number and size of plaques in their hippocampus and cerebral cortex.^43^ Also, recent studies reported that blood cadmium levels were significantly associated with AD-related mortality among older adults.^44,45^

### 2.3. Lead

Humans are exposed to lead (Pb), a significant toxic metal, through ingestion of contaminated food and water or inhalation of polluted air. Pb pollution can be caused by lead batteries, paints, electronic waste and smelting operations.^46^ The presence of Pb in the blood affects many organs but the CNS is considered the most vulnerable. Pb can compete with the binding site of metals such as calcium leading to biometal dyshomeostasis, cross the blood–brain barrier rapidly and modify neural differentiation and synaptogenesis leading to severe damage. Furthermore, Pb exposure was found to contribute to the hallmarks of AD, including Aβ accumulation, expression of tau protein, and neuroinflammation.^47^*In vitro* studies on SH-SY5Y neuroblastoma cells revealed that Pb exposure led to Aβ accumulation, APP expression and decreased mRNA and protein levels of neprilysin endopeptidase, a Aβ degrading enzyme.^48,49^ Additionally, Pb exposure in young rats increased the expression of APP and BACE1 which subsequently induced AD-like pathology by inducing Aβ accumulation and plaque formation in the hippocampus and cortex.^50^ In another *in vivo* study on rats drinking water containing 200 ppm Pb at the age of 1–20 days, a delayed overexpression of APP and elevation of its amyloidogenic Aβ product in old age was observed.^51^ Also, elevations in the oxidative DNA marker 8-hydroxy-2′-deoxyguanosine (8-oxo-dG) were reported in older rat brains that had been developmentally exposed to Pb.^52^ The first to perform primates' studies were Wu *et al.* who reported that exposure of monkeys to Pb at birth to 400 days upregulates the expression of APP and BACE1 in old age.^53^

### 2.4. Arsenic

Arsenic is a naturally occurring environmental toxicant in which contaminates the soil and drinking water and represents a global health threat. Arsenic is generally used in manufacturing lead alloys and semiconductor electronic devices. Arsenic exposure causes several health problems such as cancer, liver damage, and nervous system disturbances such as polyneuropathy, hallucinations, disorientation, and agitation.^54–56^ Arsenic has been found to be the most toxic metalloid responsible for the neurotoxicity associated with AD development in the brain, impairing cognitive functions.^57^ Such observation was supported by evidence that prolonged arsenic exposure causes an increase in tau phosphorylation and insoluble tau aggregates in SH-SY5Y cells.^58^ Another *in vitro* study showed that incubation of cholinergic SN56.B5.G4 cells with organic dimethylarsinic acid led to an increase in Aβ levels^59^ Finally, an *in vivo* study using 3xTgAD AD mice model suggested that exposure to inorganic arsenic can lead to behavioral impairments, presence of amyloid plaques and neurofibrillary tangles, thus exacerbating AD pathophysiological progression.^60^ Selected studies investigating the effects of mercury, lead, cadmium, and arsenic exposure on AD pathology are summarized in Table 1.

**Table 1:** Summary of studies on heavy metals effect on AD pathology

Metal	Study model	Outcome	References
Mercury	Rats	Mercury induces neuronal beta-tubulin defects	26 and 27
	AD patients	Mercury concentration in plasma is doubled than control group	28
	Workers with high exposure to mercury	Symptoms of cognitive deficit are witnessed	29 and 30
	Autopsied brain of AD patient	Mercury deposition in hippocampus and amygdala	31
Cadmium	Transgenic mice (APP/PS1 mice)	Cd increases Aβ-42 production and enlarges size of senile plaque	43
	AD patients	Blood cadmium levels is associated with AD morbidity rate	44 and 45
Lead	SH-SY5Y neuroblastoma cells	Pb exposure leads to Aβ secretion, APP expression and decrease in NEP	48 and 49
	Rats	Increased the expression of APP and BACE1, induce Aβ accumulation and plaque formation in the hippocampus and cortex	50
	Rats	Pb exposure at birth causes overexpression of APP and elevation of its amyloidogenic Aβ product in old age	51
	Rats	Elevations of 8-oxo-dG as a result of Pb exposure	52
	Monkeys	Pb exposure at birth lead to expression of APP and BACE1 in old age	53
Arsenic	SH-SY5Y cells	As exposure causes an increase in tau phosphorylation and insoluble tau aggregates	58
	Cholinergic SN56.B5.G4 cells	Organic dimethylarsinic acid (DMA) led to an increase in Aβ levels	59
	3xTgAD AD mice model	Inorganic arsenic can lead to behavioral impairments, presence of amyloid plaques and neurofibrillary tangles	60

## 3. Chemosensors for sensing metal ions

Recently, optical chemosensors have shown a great potential for monitoring the toxic heavy metals present in the environment.^61^ Optical chemosensors generate visual signals for qualitative detection of toxic metals as ref. 62. Other advantages of optical chemosensors include sensitivity, selectivity, low cost, fast response time, and low detection limits. Furthermore, they allow for simple and quantitative simultaneous detection of multiple toxic metals if used in conjunction with simple colorimeters which reduces the errors caused by sample storage and transportation for analyses by central sophisticated laboratory instruments such as ICP-MS. Optical chemosensors change color upon binding of their immobilized indicators (chromophores) to their target analytes.

### 3.1. Chemosensor design

**Fig. 2:**
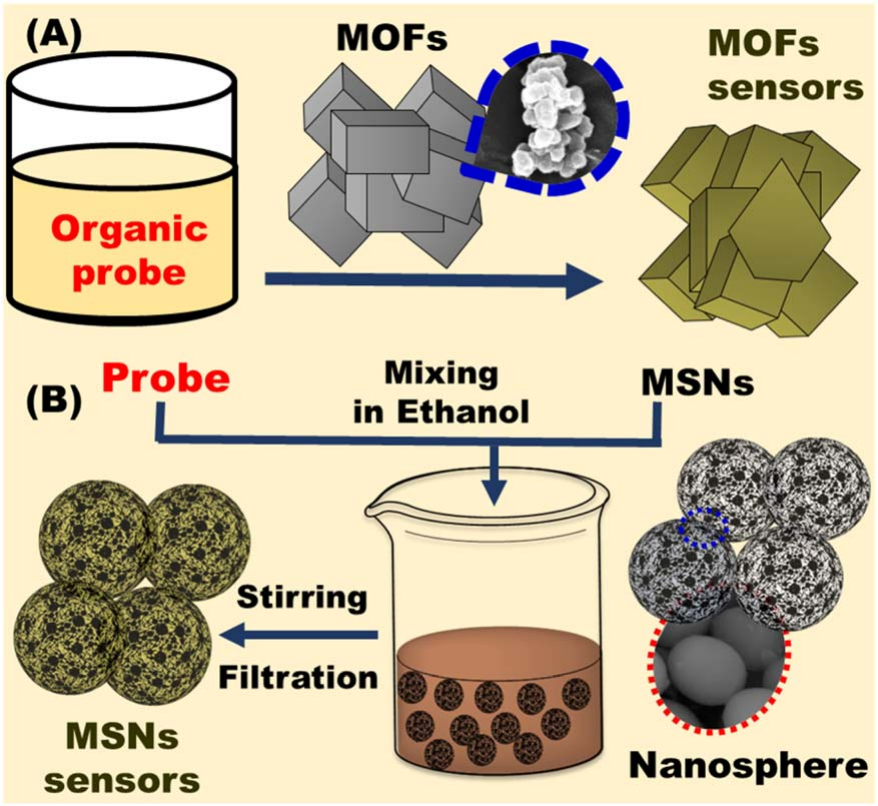
Fabrication of optical chemosensors using (A) metal organic frameworks (MOFs) and (B) mesoporous silica (MSNs).

Selecting the right carriers is critical for designing chemosensors. In general, many carriers have been used such as zeolites, metal oxides, polymers, metallosupramolecular network, and carbon-based materials.^63^ Mesoporous silica or metal organic frameworks are generally used as carriers in the preparation of optical chemosensors (Fig. 2).^64,65^ Although, some heavy metals are directly detected *via* ocular property such as luminescence or absorption, there are some targets has no absorption or luminescence property, therefore, optical chemosensors with selective and sensitive optical probe were designed. The designed chemosensor may take various forms such as powder, gels, thin films or nanoparticles. The sensor changes its optical properties in presence of toxic metals.^66^ The detection of common cations in water, cosmetic, or blood is based on complexation between the chromophore and its specific target cation. Therefore, highly selective chromophores with electron donating atoms were prepared and immobilized on carriers *via* covalent, physical, or electrostatic interactions.^67^ Proper selection of mesoporous or MOFs nanomaterials is crucial as it affects mechanical stability of the sensor, dye immobilization, and access of the toxic metals.^68^

#### 3.1.1. Nanoporous carriers

Common porous materials include porous metal oxides, mesoporous silicates, porous carbons, and porous polymers.^63^ According to their pore size, porous substance can be categorized into macroporous, mesoporous and microporous materials.^69^ Mesoporous and microporous materials act as adsorbents^70,71^ or scaffolds for loading chromophores when utilized in metal ion detection. This improves recognition of the target toxic metals and enhance the generated signal. The large surface area of these materials provides more active sites for metal adsorption. The powerful and rapid response toward metal ions can be achieved by adjusting their surface area, and the pore diameter, allowing various metal ions monitoring and a low detection limit. Mesoporous silica and metal–organic frameworks have been used to design optical chemosensors.^72,73^

##### 3.1.1.1. Mesoporous silica

**Fig. 3:**
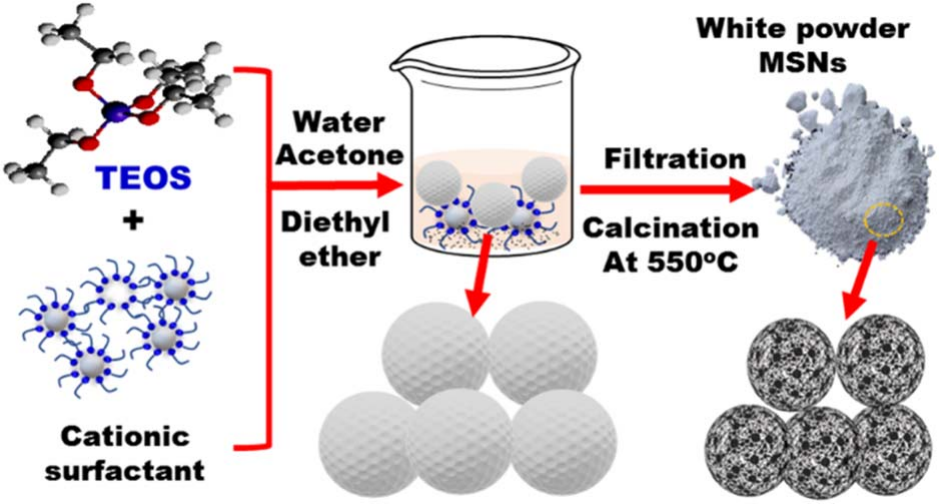
Preparation of mesoporous silica as nanoporous carrier. Briefly, TEOS (tetraethoxysilane) is reacted with a cationic surfactant (*e.g.*, CTAB) in water/acetone/diethyl ether solvent to form mesoporous silica nanospheres (MSNs). Filtration is then carried out followed by calcination at 550 °C.

The large surface area and stability of mesoporous silica nanomaterials enabled their utilization as carriers in the fields of sensing^74^ and catalysis.^75^ The soft and hard template methods were used to fabricate different structures and formation of mesoporous cavity. In the hard template approach, the rattle-type of silica nanoparticles was prepared with controllable morphology and structure. The calcination process is used for preparation of mesoporous silica as shown in Fig. 3. Therefore, the synthesis of MSN is conducted under relatively mild conditions (Fig. 3). The mesoporous nanomaterials are employed in optical chemosensors as carriers of chromophores for enhanced heavy metal recognition. To detect the selected heavy metal ions, the outer surface of MSN nanomaterials is decorated with the organic chromophore *via* physical or chemical interactions.

##### 3.1.1.2. Metal–organic frameworks

**Fig. 4:**
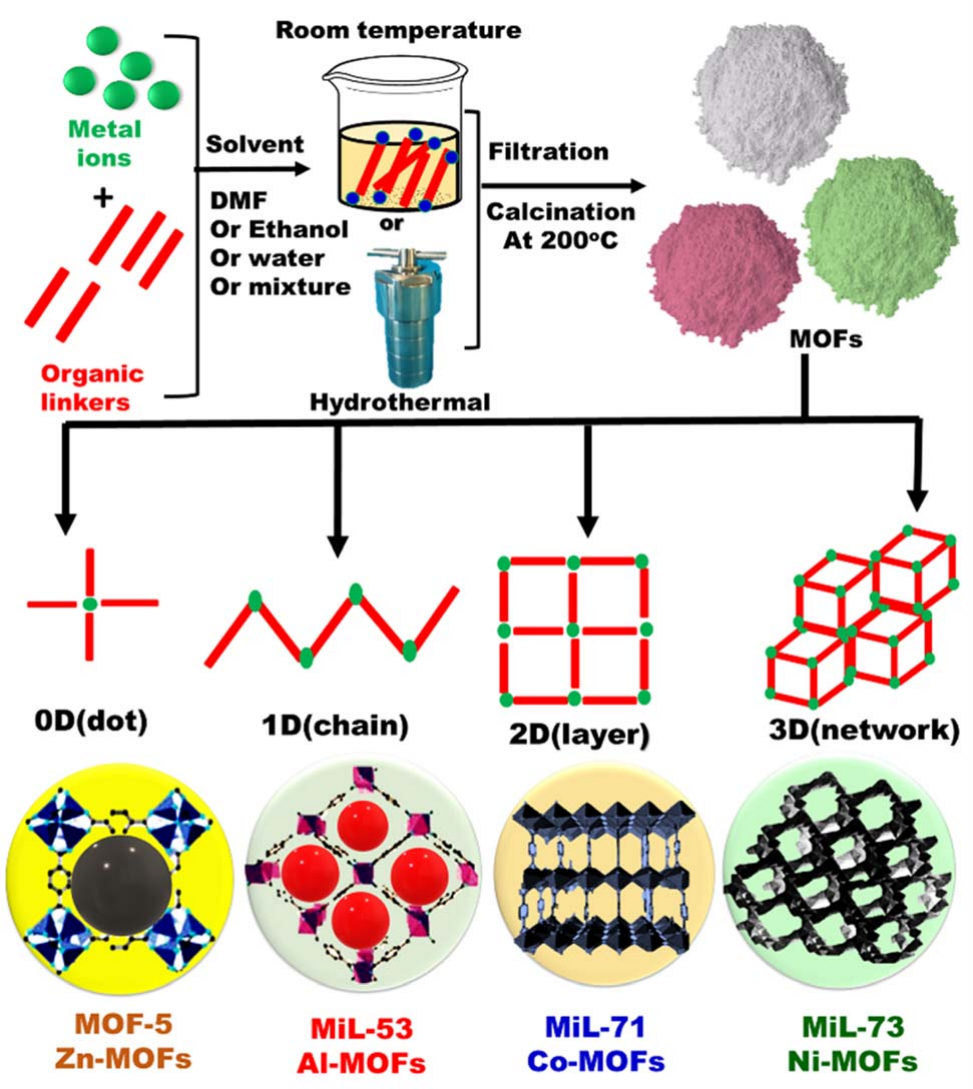
General schematic representation for fabrication of metal organic frameworks (MOFs) with different dimensions.

**Fig. 5:**
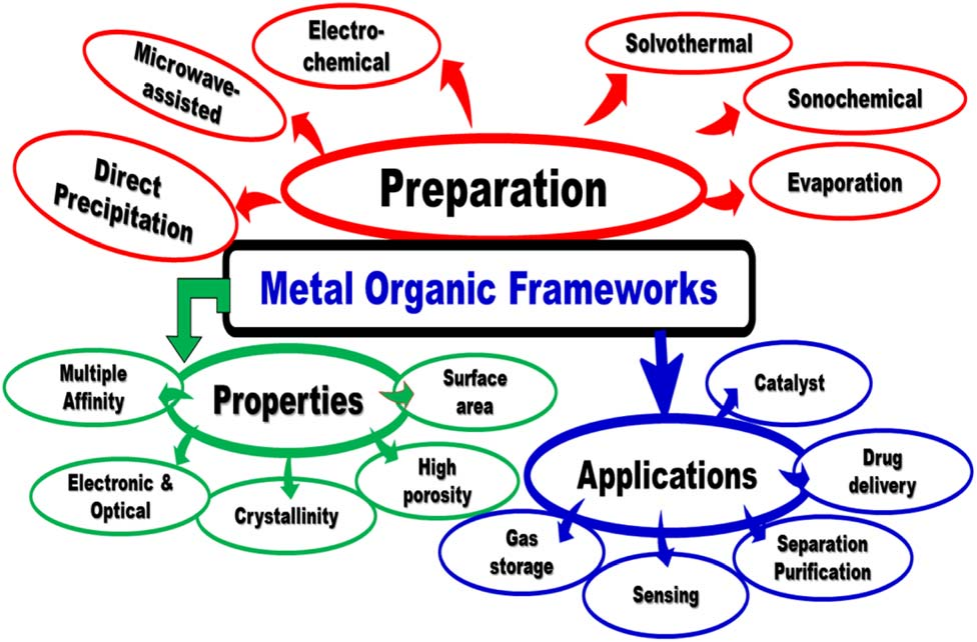
Preparation, properties and applications of metal organic frameworks.

Metal organic frameworks (MOFs) are promising hybrid porous materials, prepared by joining metal–oxygen clusters with organic linkers. MOFs consist of metal nodes linked with organic ligands *via* coordinate bond, and the ligands play a vital role in designing high specific surface areas and porous structure.^76^ Generally, the metal nodes and linkers play a significant role in the structure of MOFs (Fig. 4). The organic linkers are secondary building units (as rods) whereas the metal centers act as junctions of MOF constructions. To build up MOF structures, changing the metal centers and selecting a suitable linker are considered the main factors in designing the framework topology. The superior porosity with significant large pores over volume properties are tailored by broadened MOF's topology (Fig. 5). The substantial MOF pore volume is dependent on the organic linker size and bulky metal clusters' network. Moreover, MOFs are used for immobilizing catalysts on conductive scaffolds with high surface area and pore size. Owing to their crystallinity and porosity, the MSNs or MOFs were used as the carrier in optical chemical sensors for detection of common cations.^77,78^ MOFs materials were used for other applications such as catalysis^79^ and gas adsorption/storage.^80^

##### 3.1.1.3. Other nanoporous carriers

Several nanomaterials have been utilized as porous carriers because of their unique characteristics.^81^ Nanocrystalline TiO_2_ films were employed for colorimetric naked-eye detection of mercury in aqueous solution.^82,83^ Chey *et al.* investigated indirect determination of Hg^2+^ using ZnO nanorods.^84^ Furthermore, polymeric carriers play an important role in protecting photochromic characteristics from environmental degradation. The flat sheet cellulose acetate membrane was used as a substrate for loading dithizone chromophore for naked eye detection of Hg^2+^.^85^ Paper-based chemosensors (PBCs) were recently developed for monitoring Co^2+^ and Cd^2+^ in cosmetic products by adsorbing the chromophore onto filter papers coated with mesoporous silica.^64^

#### 3.1.2. Probe immobilization

**Fig. 6:**
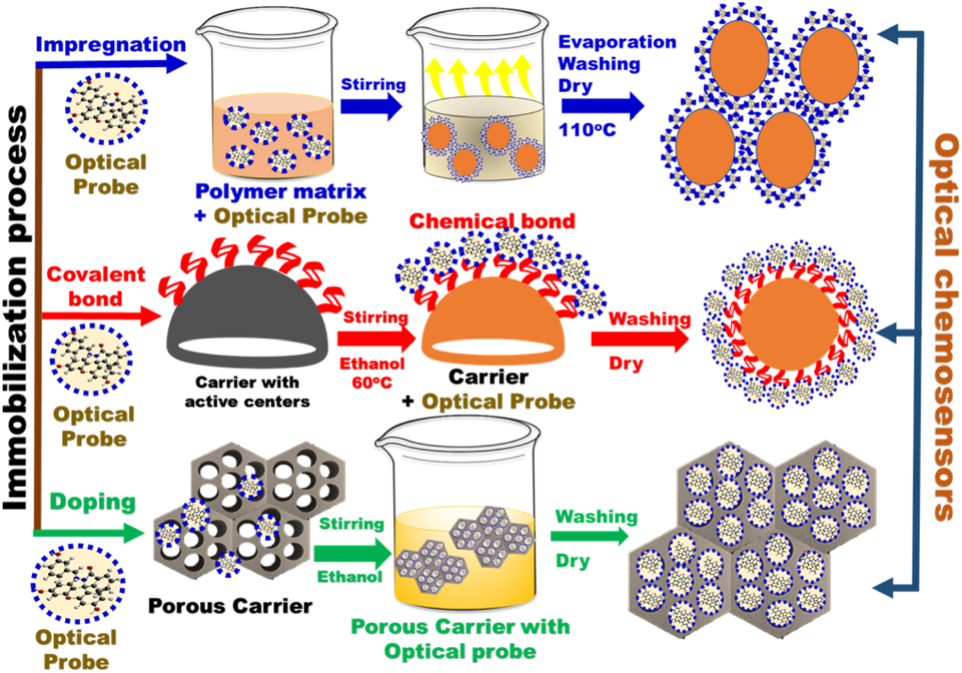
General schematic diagram of different immobilization approaches of organic probes on different carriers.

The immobilization of an organic probe onto a suitable porous material or polymer matrix, has a significant effect on the sensor monitoring features. The common approaches for immobilization of optical chemosensors include impregnation techniques, covalent bonding, and doping approach (Fig. 6).

##### 3.1.2.1. Impregnation of existing matrices

Impregnation is a classical method for loading a chromophore on a porous carrier. In this approach, a thin support film of the carrier is dipped into a saturated solution of chromophore and the solvent is left to evaporate.^86,87^ The organic chromophore is loaded into the polymer matrix *via* chemisorption or electrostatic interactions. Although the impregnation technique is inexpensive, leaching of the chromophore (due to weak bonds with the carrier) hinders the large-scale utilization of this approach.

##### 3.1.2.2. Covalent bonding to existing matrices

In this approach, the optical chromophore is chemically bonded to the surface of mesoporous or microporous materials *via* its functional groups. Alternatively, the chromophore may interact chemically with certain monomers to form a copolymer.^88,89^ Chemosensors prepared by covalent immobilization are highly stable and can be used multiple times. Disadvantages of chemosenors prepared by this method include low sensitivity and extended response time.

##### 3.1.2.3. Doping

This is the most commonly used approach for immobilization of optical probes onto nanomaterials. Significantly, the selected chromophore is introduced to the porous nanomaterial solution during the preparation process.^90–93^ An optical sensor prepared by the doping method has high stability as compared to that prepared using the impregnation technique. Moreover, the response time is better than that observed with chemosensors prepared using the covalent bonding approach.

## 4. Detection of AD related heavy metals

Optical chemosensors can be divided according to the mechanism scheme into three main categories: carrier-based ion sensing, intrinsic metal ion sensors, and indicator-mediated ion sensing.^78^

### 4.1. Mercury (Hg)

**Fig. 7:**
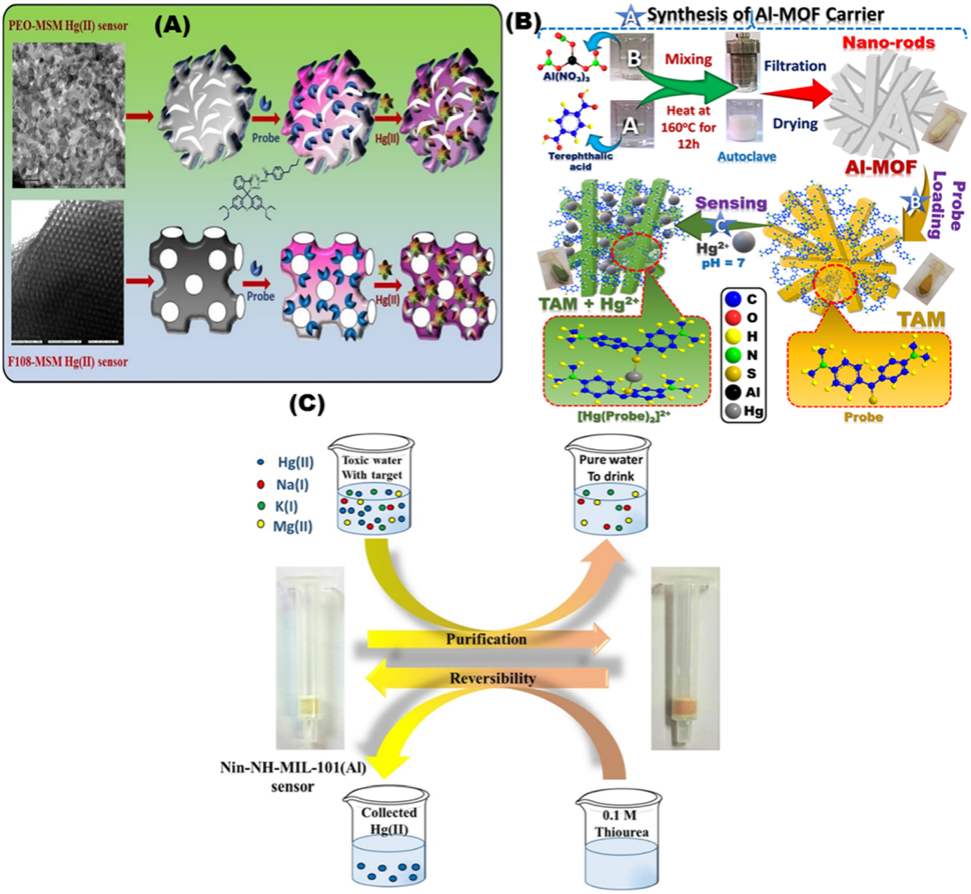
(A) Schematic diagram for sensing Hg^2+^ ions using bis(diethylamino)-3oxospiro[isoindoline-1,9′-xanthen]-2ylcarbamothioyl)-4-butylbenzamide (BOICB) probe.^94^ (B) Schematic representation of fabrication of nanorod TAM optical chemosensors and interactions with Hg^2+^ ions under optimum conditions.^95^ (C) Representative design of the Nin-NH-MIL-101(Al) sensor applied for purification of water polluted with Hg(ii) ions and the reversible process by using 0.1 M thiourea solution (which can be repeated several times).^96^ Reproduced with permission from ref. ^94–96^.

Kongasseri and co-workers reported the fabrication of sensor for the quantification of toxic Hg^2+^ ions through sol–gel process using two different block-polymer surfactants (PEO and F108) *via* doping method. The low limit of detection for probe anchored F108-MSM in sensing of mercury ions was 0.61 and 2.05 ppb; respectively (Fig. 7A). The sensor can be reused over 12 times for six months of storage.^94^ Radwan *et al.*, used optical chemosensors *via* impergnation method of bis(4-(dimethylamino)phenyl) methanethione into Al-MOFs for sensing Hg^2+^ in water samples and skin whitening products.^95^ The sensitivity and adsorption capacity of optical chemosensors for Hg^2+^ were 0.8 ppb and 1110 mg g^−1^; respectively (Fig. 7B). The Hg^2+^ sensor was used after multiple regeneration/reuse cycles (>9 cycles). Simple optical sensor for monitoring and removal of Hg^2+^ from aqueous media using amino-functionalized MOF with ninhydrin was designed by Shahat and co-workers using covalent bonding approach.^96^ They detected Hg^2+^ with a low limit of detection of ∼0.494 ppb in water and the sensor can be for (≥6) cycles (Fig. 7C).

### 4.2. Cadmium (Cd)

**Fig. 8:**
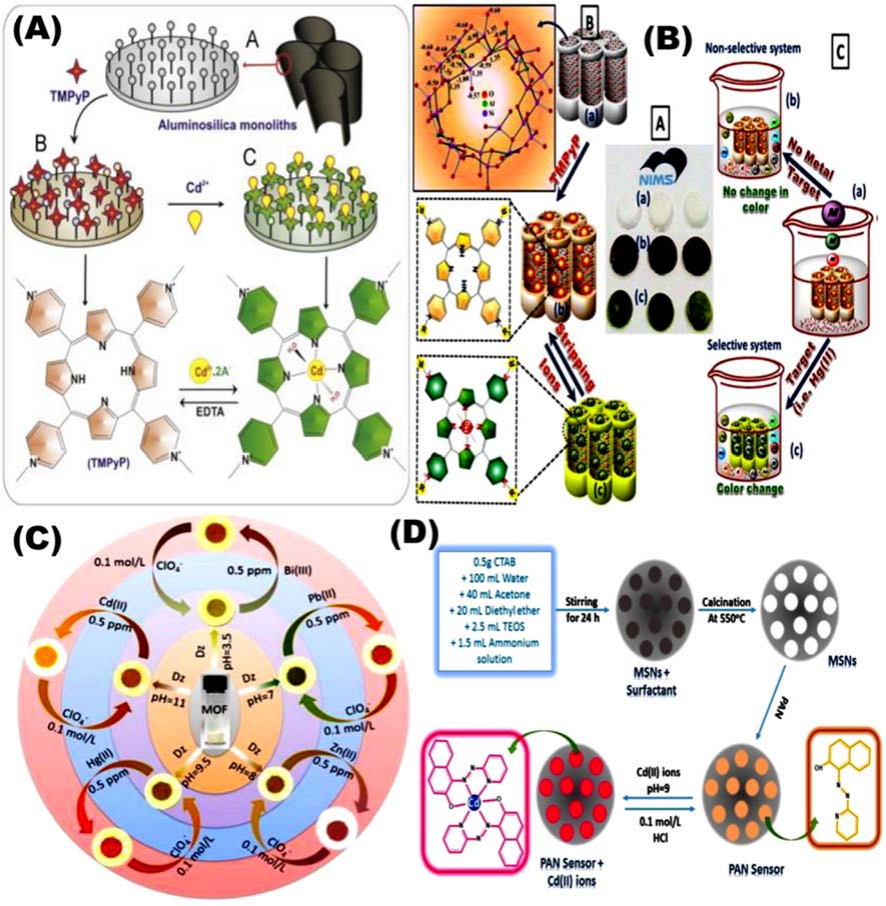
(A) General schematic presentation of optical chemosensors based on aluminosilica network platforms *via* direct functionalization with δ-tetrakis(1-methylpyridinium-4-yl)porphine ρ-toluenesulfonate for Cd(ii) ions detection.^76^ (B) Chelating ligand immobilized mesostructures Ia 3d aluminosilica for visualization and removal of Cd(ii).^97^ (C) The Zr-based metal–organic frameworks (UiO-66) with its micropore geometry for the visual detection, determination and removal of ultra-trace of some toxic metal ions such as Cd^2+^.^98^ (D) General steps of fabricating Cd^2+^ ion optical chemosensors built on mesoporous nanosphere silica for naked-eye determination of ultra-traces of Cd^2+^ ions.^99^ Reproduced with permission from ref. ^69,97–99^.

Aluminosilica-based network platforms were used as carriers in designing optical sensors *via* direct impregnation of TMPyP moieties without any surface modification. The optical sensor was used for detection and removal of Cd^2+^ ions at low concentrations (10^−10^ mol dm^−3^) for different analytical applications (Fig. 8A).^69^ Shenashen *et al.*, used aluminosilica carrier to immobilize chromophore for Cd^2+^ ions detection using impregnation approach (Fig. 8B). This optical chemosensor was utilized for removal and visualization of some toxic metals such as Cd^2+^ at (∼10^−11^ mol dm^−3^) in water and a suspension of red blood cells (RBCs) and could be regenerated/reused for 6 cycles.^97^ Shahat and co-workers developed an optical sensor based on Zr-MOFs (UiO-66) for detection, and removal of Cd^2+^ ions. The UiO-66 was used for impregnation of dithizone (Fig. 8C) without any coupling agent. The reported detection limit was 10^−10^ mol dm^−3^ and could be reused for 6 times.^98^ Radwan *et al.*, designed optical chemosensors *via* using impregnation technique of the 1-(2-pyridylazo)-2-naphthol with the mesoporous cavities of nanospheres silica for visual detection of Cd^2+^ in water samples. Digital image analysis was applied to determine the Cd^2+^ concentration in well water samples with a low detection limit of 10^−9^ mol L^−1^. The sensor could be stored for more than 8 months and reused for (*i.e.* ≥9) cycles.^99^

### 4.3. Lead (Pb)

**Fig. 9:**
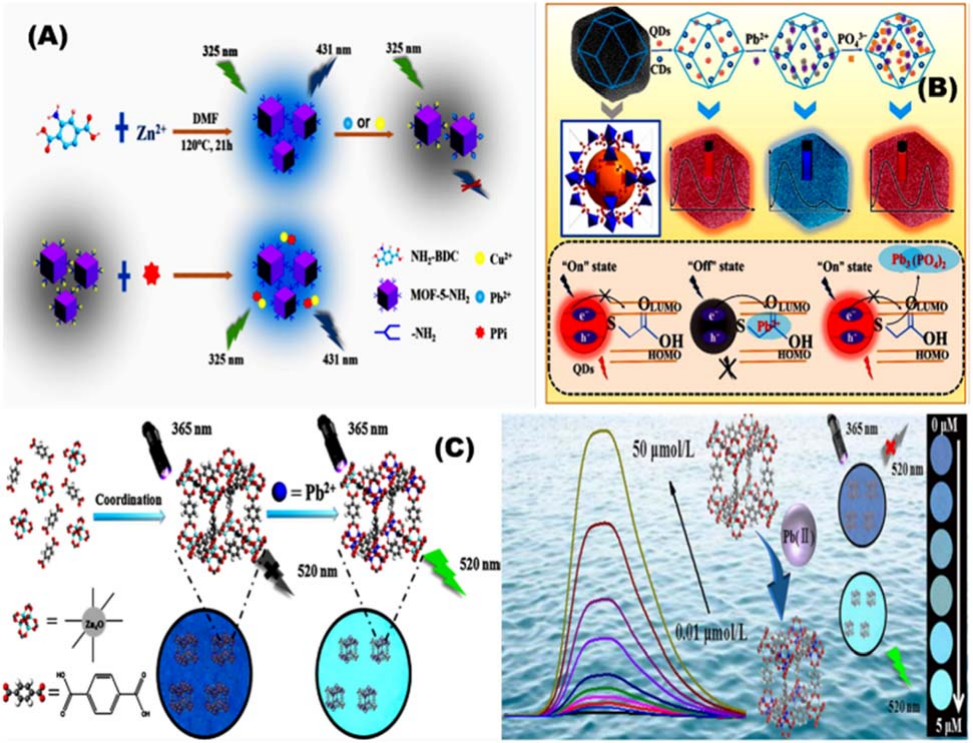
(A) General representation of fluorescent probe prepared based on amino-functionalized metal–organic frameworks (MOF-5-NH_2_) for the detection of Pb^2+^.^100^ (B) Ratiometric fluorescent (RF) probe CDs/QDs@ZIF-8 by encapsulating carbon dots (CDs) and thioglycolic acid modified CdTe quantum dots (QDs) into porous metal–organic frameworks (MOFs) for detection of Pb^2+^.^101^ (C) Phosphorescent sensor to monitor Pb^2+^ using metal–organic framework-5.^102^ Reproduced with permission from ref. ^100–102^.

Xuanxuan and coworkers prepared fluorescent probe based on amino-functionalized MOFs (MOF-5-NH_2_) using covalent bonding approach for sensing of Pb^2+^ using a single step synthesis (Fig. 9A). The Pb^2+^ coordinated with the amino groups on the surface of the MOF-5-NH_2_ thus allowing for fluorescence quenching with a low limit of detection of 0.25 μmol L^−1^.^100^ Kuiyu Yi and Lei Zhang designed fluorescence probe by encapsulating thioglycolic acid modified CdTe quantum dots (QDs) and carbon dots (CDs) into porous (MOFs) for detection of Pb^2+^ using doping method in biological samples (Fig. 9B).^101^ Shifen Xua and coworkers developed Pb^2+^ phosphorescent chemosensors using MOF-5. The Pb^2+^ chemosensors showed a low detection limit of 2 nmol L^−1^ and linear range of 0.01–10 μmol L^−1^ (Fig. 9C). They prepared phosphorescent chemosensors in a polyethylene glycol film using impregnation method, which exhibit color change at 1.0 μmol /L^−1^ Pb^2+^ under 365 nm UV light.^102^

### 4.4. Arsenic

Biswajit *et al.* reported a carboxylate-rich MOF for As(iii) detection where a linear increase in the fluorescence intensity was observed for arsenic concentration in the range of 3.67–332 ng L^−1^.^103^ In another work, a MOF functionalized nanoconjugate material was prepared by Shahat *et al.* for selective sensing of As(v) and phosphate in the aqueous media. The detection limits of As(v) and phosphate were 0.15 μg L^−1^ and 0.13 μg L^−1^, respectively. Detection was observed *via* change of the MOF color to blue, which was analyzed by UV-vis spectroscopic techniques.^104^ Different chemosensors were utilized for detection of mercury, lead, cadmium, and arsenic were listed in Table 2.

**Table 2:** Chemosensors used for detection of mercury, cadmium, lead, and arsenic ions

Metal	Chemosensors	Substrate	Probe mechanism	Detection limit (μM)	Ref.
Hg	Probe anchored F108-MSM	MSNs	Absorbance	0.003	94
	Bis(4(dimethylamino)phenyl)methanethione into Al-mofs	MOFs	Absorbance	0.0039	95
	Amino-functionalized MOF with ninhydrin	MOFs	Absorbance	0.0024	96
Cd	Aluminosilica-based network platforms	Aluminosilica	Absorbance	0.03	69
	A porphyrinic chelating ligand	Aluminosilica	Absorbance	0.03	97
	Optical sensor based on Zr-mofs (uio-66)	MOFs	Absorbance	0.18	98
	1-(2-Pyridylazo)-2-naphthol with the mesoporous silica	MSNs	Absorbance	0.034	99
Pb	Amino-functionalized MOFs (MOF-5-NH_2_)	MOFs	Fluorescence	0.25	100
	CdTe quantum dots (QDs) and carbon dots (CDs) into porous (MOFs)	MOFs	Fluorescence	0.0235	101
	Phosphorescent chemosensors using MOF-5	MOFs	Phosphorescence	0.02	102
As	Carboxylate-rich MOF	MOFs	Fluorescence	0.049	103
	MOF functionalized nanoconjugate material	MOFs	Absorbance	0.15	104

## 5. Challenges in the field of optical sensors

Although qualitative results generated by optical chemosensors are useful to identify contaminated water sources, quantitative results are needed to guide actions based on guideline cut off values. Since the basic idea behind the development of chemosensors is the specificity of the signal obtained, it is important to implement selective recognition moieties (probes) for the target metal. Adjusting the detection conditions (such as temperature, pH, sensor concentration, and contact time) is critical for optimal detection. The ideal optical chemosensor should have high selectivity, sensitivity, low detection limit, and a short response time. It should also be reusable, inexpensive, and simple to use. Optical chemosensors have been used for sensing and monitoring of toxic metals and radioactive isotopes and bioactive species.

## 6. Conclusions and future perspectives

Exposure to heavy metals such as Hg, Cd, Pb and As poses a serious threat to the nervous system and may contribute to the onset and progression of AD. Therefore, monitoring environmental exposure to heavy metals is critical. Monitoring protocols based on nanoporous metal–organic framework and mesoporous silica carriers have been developed for detection of heavy metals. Rapid improvements in the nanoporous materials presented significant opportunities to fabricate novel sensors for different monitoring applications. These sensors can be used in different environments where the hazardous materials are produced or accumulate. The fabrication of optical chemosensors with exceptional structures and morphologies led to the progress of various detection techniques of ultra-trace concentrations of heavy metals. In conclusion, optical chemosensors, including those with hieratical nanochannels, could play a major role in large scale monitoring of exposure to water contaminated with AD-related heavy metals.

## Conflicts of interest

Prof. Hassan Azzazy is an inventor on a granted patent on development of chemosensors for detection of toxic metals. Other authors declare no conflict of interest.

## Supplementary Material

RA-012-D2RA05384E-s001
