# Supplementary material for: Optical chemosensors for environmental monitoring of toxic metals related to Alzheimer's disease
Source: RSC Adv. 2022 Nov 15;12(50):32744–55. doi: 10.1039/d2ra05384e (PMC9664454; doi:10.1039/d2ra05384e)
Supplement: RA-012-D2RA05384E-s001 [file RA-012-D2RA05384E-s001.pdf]

Electronic Supplementary Material (ESI) for RSC Advances.
This journal is © The Royal Society of Chemistry 2022

Supporting information

Optical Chemosensors for environmental monitoring of toxic metals related to Alzheimer's Disease.

Islam M. El-Sewify [1,2], Ahmed Radwan [1,2], Nehal H. Elghazawy[2], Wolfgang Fritzsche[3], Hassan M. E. Azzazy [2,3*]

[1]Department of Chemistry, Faculty of Science, Ain Shams University, 11566, Abbassia, Cairo, Egypt

[2]Department of Chemistry, School of Sciences & Engineering, The American University in Cairo, SSE, Rm #1194, P.O. Box 74, New Cairo 11835, Egypt

[3]Department of Nanobiophotonics, Leibniz Institute for Photonic Technology, Jena 07745, Germany

Corresponding Author
Hassan Azzazy; Department of Chemistry, School of Sciences & Engineering
The American University in Cairo, SSE, Rm #1194, P.O. Box 74, New Cairo 11835, Egypt
Tel: +202 2615 2559
E-mail: hazzazy@aucegypt.edu

Table S1. Drinking water quality guidelines (μg L^{-1}) for heavy metals

Heavy Metal	Substrate	WHO	EU	US EPA	Oxidation States	Health Effects
**Arsenic**	As	10	10	10	III, V	Skin damage, renal system failure, cancer [1,2].
**Cadmium**	Cd	3	5	5	II	Cardiovascular issues, osteoporosis, cancer [3].
**Lead**	Pb	10	10	15	II	Nervous system impairment, brain damage, cancer [4,5].
**Mercury**	Hg	1	1	2	I, II	Renal failure, neurological disorders, cancer [6,7].

Note: Reprinted from " A Review of Microfluidic Detection Strategies for Heavy Metals in Water" by Lace, A. and Cleary, J., 2021, Chemosensors, 9, 60.

References

1. B.K. Mandal, K.T. Suzuki, Arsenic round the world: A review. Talanta 58, (2002)201–235.
2. Kapaj, S.; Peterson, H.; Liber, K.; Bhattacharya, P. Human health effects from chronic arsenic poisoning—A review. J. Environ. Sci. Health Part A 41(2006)2399–2428.
3. Rahimzadeh, M.R.; Rahimzadeh, M.R.; Kazemi, S.; Moghadamnia, A.A. Cadmium toxicity and treatment: An update. Casp. J. Intern. Med. 8(2017)135.
4. M.R. Asgharipour, A.R. Sirousmehr, Comparison of three techniques for estimating phytotoxicity in municipal solid waste compost. Ann. Biol. Res., 3(2012)1094–1101.
5. M. Vilar, J. Barciela, S. García-Martín, R.M. Pena, C. Herrero, Comparison of different permanent chemical modifiers for lead determination in Orujo spirits by electrothermal atomic absorption spectrometry. Talanta, 71(2007)1629–1636.
6. H. Satoh, Occupational and environmental toxicology of mercury and its compounds. Ind. Health, 38(2000)153–164.
7. J.F. Risher, S.N. Amler, Mercury exposure: Evaluation and intervention: The inappropriate use of chelating agents in the diagnosis and treatment of putative mercury poisoning. Neurotoxicology, 26(2005)691–699.
